# Supplementary material for: Novel Driver Strength Index highlights important cancer genes in TCGA PanCanAtlas patients
Source: PeerJ. 2022 Aug 11;10:e13860. doi: 10.7717/peerj.13860 (PMC9375969; doi:10.7717/peerj.13860)
Supplement: Supplemental Information 2 [file peerj-10-13860-s002.zip › COHORTS/genes plots/stage/2021_8_16_14_9_distribution_stage_females_II.pdf]

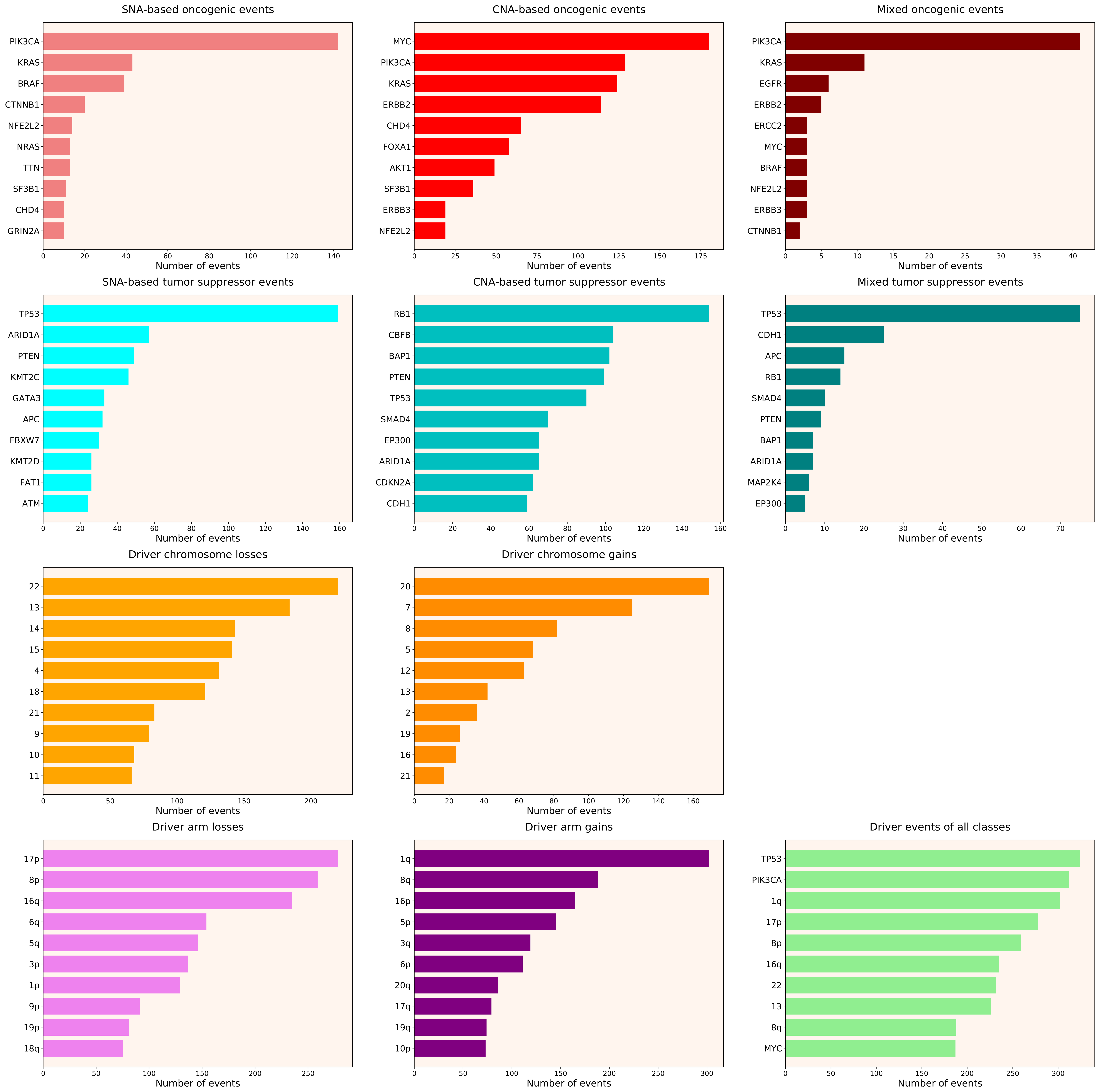

SNA-based oncogenic events
PIK3CA
KRAS
BRAF
CTNNB1
NFE2L2
NRAS
TTN
SF3B1
CHD4
GRIN2A
0 20 40 60 80 100 120 140
Number of events
CNA-based oncogenic events
MYC
PIK3CA
KRAS
ERBB2
CHD4
FOXA1
AKT1
SF3B1
ERBB3
NFE2L2
0 25 50 75 100 125 150 175
Number of events
Mixed oncogenic events
PIK3CA
KRAS
EGFR
ERBB2
ERCC2
MYC
BRAF
NFE2L2
ERBB3
CTNNB1
0 5 10 15 20 25 30 35 40
Number of events
SNA-based tumor suppressor events
TP53
ARID1A
PTEN
KMT2C
GATA3
APC
FBXW7
KMT2D
FAT1
ATM
0 20 40 60 80 100 120 140 160
Number of events
CNA-based tumor suppressor events
RB1
CBFB
BAP1
PTEN
TP53
SMAD4
EP300
ARID1A
CDKN2A
CDH1
0 20 40 60 80 100 120 140 160
Number of events
Mixed tumor suppressor events
TP53
CDH1
APC
RB1
SMAD4
PTEN
BAP1
ARID1A
MAP2K4
EP300
0 10 20 30 40 50 60 70
Number of events
Driver chromosome losses
22
13
14
15
4
18
21
9
10
11
0 50 100 150 200
Number of events
Driver chromosome gains
20
7
8
5
12
13
2
19
16
21
0 20 40 60 80 100 120 140 160
Number of events
Driver arm losses
17p
8p
16q
6q
5q
3p
1p
9p
19p
18q
0 50 100 150 200 250
Number of events
Driver arm gains
1q
8q
16p
5p
3q
6p
20q
17q
19q
10p
0 50 100 150 200 250 300
Number of events
Driver events of all classes
TP53
PIK3CA
1q
17p
8p
16q
22
13
8q
MYC
0 50 100 150 200 250 300
Number of events
